# Mutations in *TAC1B*: a Novel Genetic Determinant of Clinical Fluconazole Resistance in Candida auris

**DOI:** 10.1128/mBio.00365-20

**Published:** 2020-05-12

**Authors:** Jeffrey M. Rybak, José F. Muñoz, Katherine S. Barker, Josie E. Parker, Brooke D. Esquivel, Elizabeth L. Berkow, Shawn R. Lockhart, Lalitha Gade, Glen E. Palmer, Theodore C. White, Steve L. Kelly, Christina A. Cuomo, P. David Rogers

**Affiliations:** a Department of Clinical Pharmacy and Translational Science, University of Tennessee College of Pharmacy, Memphis, Tennessee, USA; b Broad Institute of MIT and Harvard, Cambridge, Massachusetts, USA; c Centre for Cytochrome P450 Biodiversity, Institute of Life Science, Swansea University Medical School, Swansea, United Kingdom; d School of Biological Sciences, University of Missouri at Kansas City, Kansas City, Missouri, USA; e Mycotic Diseases Branch, Centers for Disease Control and Prevention, U.S. Department of Health and Human Services, Atlanta, Georgia, USA; Tel Aviv University

**Keywords:** *Candida*, triazole, resistance, efflux, CRISPR, WGS, antifungal resistance, drug efflux

## Abstract

Candida auris has emerged as a multidrug-resistant pathogen of great clinical concern. Approximately 90% of clinical C. auris isolates are resistant to fluconazole, the most commonly prescribed antifungal agent, and yet it remains unknown what mechanisms underpin this fluconazole resistance. To identify novel mechanisms contributing to fluconazole resistance in C. auris, fluconazole-susceptible C. auris clinical isolate AR0387 was passaged in media supplemented with fluconazole to generate derivative strains which had acquired increased fluconazole resistance *in vitro*. Comparative analyses of comprehensive sterol profiles, [^3^H]fluconazole uptake, sequencing of C. auris genes homologous to genes known to contribute to fluconazole resistance in other species of *Candida*, and relative expression levels of C. auris *ERG11*, *CDR1*, and *MDR1* were performed. All fluconazole-evolved derivative strains were found to have acquired mutations in the zinc-cluster transcription factor-encoding gene *TAC1B* and to show a corresponding increase in *CDR1* expression relative to the parental clinical isolate, AR0387. Mutations in *TAC1B* were also identified in a set of 304 globally distributed C. auris clinical isolates representing each of the four major clades. Introduction of the most common mutation found among fluconazole-resistant clinical isolates of C. auris into fluconazole-susceptible isolate AR0387 was confirmed to increase fluconazole resistance by 8-fold, and the correction of the same mutation in a fluconazole-resistant isolate, AR0390, decreased fluconazole MIC by 16-fold. Taken together, these data demonstrate that C. auris can rapidly acquire resistance to fluconazole *in vitro* and that mutations in *TAC1B* significantly contribute to clinical fluconazole resistance.

## Abstract (precis)

Candida auris is an emerging multidrug-resistant pathogen of global concern, known to be responsible for outbreaks on six continents and to be commonly resistant to antifungals. While the vast majority of clinical C. auris isolates are highly resistant to fluconazole, an essential part of the available antifungal arsenal, very little is known about the mechanisms contributing to resistance. In this work, we show that mutations in the transcription factor *TAC1B* significantly contribute to clinical fluconazole resistance. These studies demonstrated that mutations in *TAC1B* can arise rapidly *in vitro* upon exposure to fluconazole and that a multitude of resistance-associated *TAC1B* mutations are present among the majority of fluconazole-resistant C. auris isolates from a global collection and appear specific to a subset of lineages or clades. Thus, identification of this novel genetic determinant of resistance significantly adds to the understanding of clinical antifungal resistance in C. auris.

## INTRODUCTION

First identified in 2009, Candida auris has rapidly become a health care-associated and multidrug-resistant pathogen of global concern (1, 2). While originally found to be the causative pathogen of virtually simultaneous outbreaks of invasive candidiasis in Asia, South Africa, and South America, C. auris has now been identified in more than 30 countries across 6 continents, including more than 900 confirmed clinical cases of C. auris infections in the United States (3). Further contributing to the clinical significance of this organism are its proclivity to colonize both environmental surfaces and patients, challenges associated with reliable identification in the clinical microbiology laboratory, and the markedly decreased susceptibility to currently available antifungal agents found in a large proportion of C. auris clinical isolates (4, 5). While epidemiological data and clinical experience pertaining to the treatment of infections caused by C. auris are currently inadequate to support the establishment of epidemiological cutoff values and true clinical breakpoints, the Centers for Disease Control and Prevention (CDC) has proposed tentative breakpoints to help guide clinicians on the basis of available susceptibility data for C. auris clinical isolates. Applying these tentative breakpoints, approximately 3% of C. auris clinical isolates are resistant to echinocandins, one-third are resistant to amphotericin B, and 90% are resistant to fluconazole (MIC, ≥32mg/liter; modal MIC, ≥256mg/liter) (6). Additionally, one-third of clinical isolates show multidrug resistance, with elevated MIC levels for agents from two or more different classes of antifungals, and clinical isolates resistant to all available agents have been repeatedly reported (7, 8).

The extent of fluconazole resistance among C. auris isolates is particularly concerning as this agent remains the most commonly prescribed antifungal, and many of the outbreaks of C. auris have occurred in resource-limited settings (2, 8–11). While the pervasiveness of fluconazole resistance among C. auris clinical isolates substantially limits therapeutic options of C. auris infections, relatively little is known about the molecular mechanisms underpinning this resistance. One mechanism of fluconazole resistance repeatedly identified in C. auris is mutation of the gene encoding the sterol-demethylase enzyme targeted by the triazoles, *ERG11.* Three such mutations, encoding the amino acid substitutions VF125AL (commonly referred to as F126L), Y132F, and K143R, are frequently reported among fluconazole-resistant clinical isolates, and associations between these mutations and specific genetic clades of C. auris have been observed (2). Additionally, the mutations encoding the Y132F and K143R substitutions correspond to mutations known to contribute to triazole resistance in other species of *Candida* such as Candida albicans (12). While the direct impact of these *ERG11* mutations has not been delineated in C. auris, heterologous expression of C. auris *ERG11* alleles carrying mutations encoding either the Y132F or K143R amino acid substitution on a low-copy-number episomal plasmid was observed to decrease fluconazole susceptibility in a haploid strain of Saccharomyces cerevisiae (13). However, clinical isolates harboring the same *ERG11* mutations and exhibiting a fluconazole MIC as low as 1 mg/liter have been described previously, as have fluconazole-resistant isolates of C. auris with no mutation in *ERG11*, suggesting the presence of yet-to-be-identified mechanisms of fluconazole resistance (8, 14).

In addition to mutations in *ERG11*, increased expression of efflux pump-encoding genes is a common contributor to clinical triazole resistance among multiple species of *Candida* (15). Most notable of these is C. glabrata, in which nearly all of the clinical triazole resistance is attributable to overexpression of the ATP-binding cassette (ABC)-type efflux pump-encoding genes C. glabrata CDR1 (*CgCDR1*), *CgPDH1*, and *CgSNQ2* (16). The C. auris genome has recently been revealed to include a substantial number of efflux pump-encoding genes of both the ABC and major facilitator superfamily (MFS) classes, and triazole-resistant isolates of C. auris have been observed to exhibit efflux pump activity greatly exceeding (up to 14-fold higher) that of C. glabrata (17–19). Furthermore, the increased expression of the C. auris ABC-type efflux pump-encoding gene *CDR1* has previously been shown to substantially contribute to clinical triazole resistance (20, 21). At present, however, the genetic determinants underpinning the increased expression of efflux pump-encoding genes in C. auris remain unidentified.

In this work, we took an unbiased approach utilizing *in vitro* evolution to create a collection of isogenic C. auris strains with increased fluconazole resistance, exhibiting an 8-to-64-fold increase in fluconazole MIC. Characterization of these strains and analysis of whole-genome sequencing data for over 300 globally distributed C. auris isolates implicated *TAC1B* (B9J08_004820), a close homolog of the well-characterized C. albicans transcriptional regulator *CaTAC1*, as a novel genetic determinant of clinical fluconazole resistance. Having identified *TAC1B* mutations to be present among a large proportion of fluconazole-resistant clinical isolates, we utilized a Cas9-mediated transformation system both to introduce the most common *TAC1B* mutation identified among resistant clinical isolates (encoding A640V) into the fluconazole-susceptible AR0387 and to correct the A640V-encoding mutation in the previously characterized and highly fluconazole-resistant AR0390 clinical isolate to the wild-type (WT) sequence. In both cases, the presence of this prevalent *TAC1B* mutation was found to be associated with significant increase in fluconazole MIC, demonstrating that mutations in *TAC1B* represent prevalent and significant genetic determinants of fluconazole resistance among clinical C. auris isolates.

## RESULTS

### Candida auris rapidly acquires increased fluconazole resistance *in vitro*.

**FIG 1:**
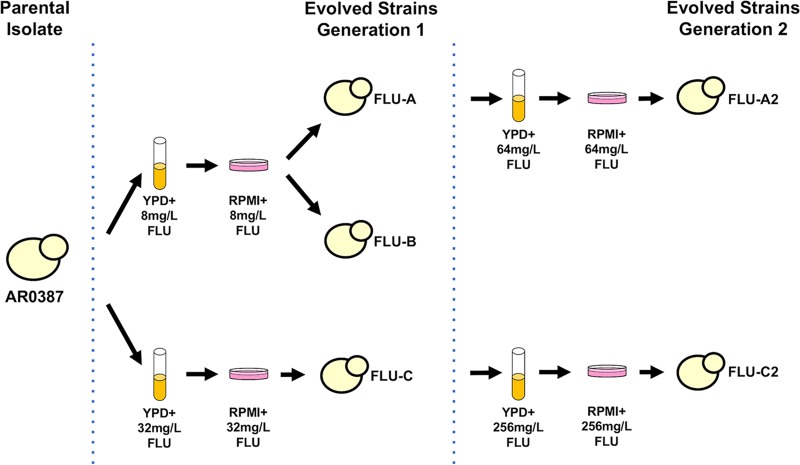
Schematic of C. auris fluconazole *in vitro* evolution experiments. To obtain fluconazole-evolved C. auris strains, AR0387 was cultured in YPD supplemented with 8 or 32 mg/liter of fluconazole. Cultures were plated on RPMI media containing the same concentration of fluconazole, and individual colonies were picked for further characterization. Fluconazole-evolved strains FLU-A and FLU-C were subsequently further passaged in YPD supplemented with 64 and 256 mg/liter of fluconazole, respectively. Cultures were then again plated on RPMI media containing the same concentration of fluconazole, and individual colonies were picked for further characterization.

In an effort to identify novel mechanisms of fluconazole resistance in this emerging multidrug-resistant pathogen, a collection of isogenic strains with increased fluconazole resistance was created via *in vitro* evolution utilizing the previously described fluconazole-susceptible C. auris clinical isolate AR0387 (also known as B8441) (Fig. 1). Briefly, the parental AR0387 was grown in liquid cultures of yeast extract-peptone-dextrose (YPD) media supplemented with either 8 or 32 mg/liter of fluconazole for 48 h. Each liquid culture was then plated on the standard antifungal susceptibility testing medium, RPMI 1640, supplemented with the same concentration of fluconazole for an additional 48 h to identify individual colonies exhibiting increased fluconazole resistance. Two individual colonies were randomly selected for characterization from the plate supplemented with 8 mg/liter of fluconazole (yielding strains FLU-A and FLU-B), and a single colony was randomly selected from the plate supplemented with 32 mg/liter of fluconazole (yielding strain FLU-C). Two strains (including one strain each from the initial passages), FLU-A and FLU-C, were subsequently subjected to a second passage in 64 and 256 mg/liter of fluconazole supplemented media, respectively, yielding strains FLU-A2 and FLU-C2.

**FIG 2:**
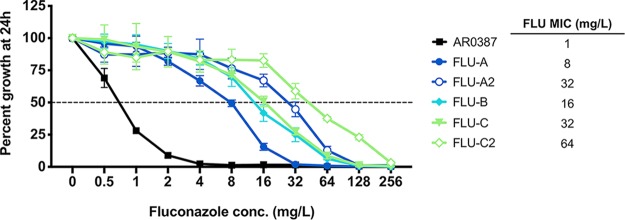
Elevated fluconazole MIC observed among C. auris fluconazole-evolved strains. Percent growth of AR0387 and fluconazole-evolved strains with escalating concentrations of fluconazole was measured at 24 h. Percent growth was determined relative to respective untreated controls as assessed by absorbance at OD_600_. Growth inhibition of 50% relative to untreated control is shown as a dotted horizontal line. The fluconazole MIC for each isolate or strain is shown at the right. Error bars for each data point represent the standard deviations of results from three independent measurements of technical replicates.

Fluconazole MICs were then determined for the parental AR0387 and each of the five fluconazole-evolved strains by broth microdilution in accordance with Clinical and Laboratory Standards Institute methodology with minor modifications as previously described (20). AR0387 exhibited a fluconazole MIC of 1 mg/liter, while the five fluconazole-evolved strains were found to have MICs ranging from 8 to 64 mg/liter (Fig. 2). Each of the second-generation evolved strains, FLU-A2 and FLU-C2, exhibited a further 2-to-4-fold increase in fluconazole MIC relative to the corresponding first-generation strains. Fluconazole MICs for all fluconazole-evolved strains were observed to be stable following storage at –80°C and multiple passages on fluconazole-free media.

### Fluconazole-evolved strains exhibit alterations in membrane sterols without accompanying mutations in *ERG11* or *ERG3*.

As fluconazole-resistant C. auris clinical isolates are very often found to possess mutations in *ERG11*, sequencing of the *ERG11* allele for each of the fluconazole-evolved strains was performed. Surprisingly, all evolved strains were found to have wild-type *ERG11* sequences matching that of the parental AR0387. To assay for other changes to the ergosterol biosynthesis pathway which may have been contributing to fluconazole resistance, each of the fluconazole-evolved strains and the parental AR0387 were subsequently subjected to comprehensive sterol profiling. Briefly, each strain was grown to the exponential-growth phase in RPMI liquid media with or without 16 mg/liter of fluconazole (a concentration approximating the average serum concentration achieved in patients being treated for candidemia) (22, 23).

**FIG 3:**
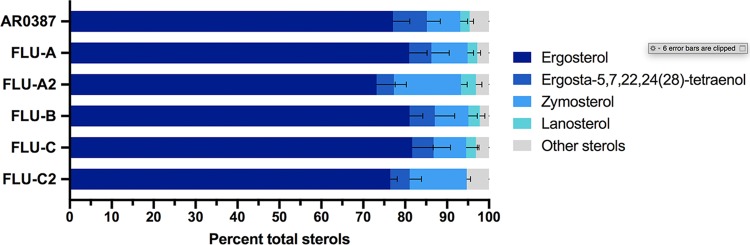
Sterol profiles of C. auris fluconazole-evolved strains grown in RPMI media are similar to those of the parental AR0387. The major constituent sterols for AR0387 and fluconazole-evolved strains at the exponential-growth phase in RPMI media are shown as a proportion of total cellular sterols. Error bars for each data point represent the standard deviations of results from three independent measurements of technical replicates.

Following growth in RPMI media without fluconazole, all of the fluconazole-evolved strains and the parental AR0387 were observed to have largely similar sterol profiles (Fig. 3). In all samples, ergosterol comprised more than 75% of total cellular sterols, with ergosta-5,7,22,24(28)-tetraenol and zymosterol observed to be the next most abundant sterols. AR0387 and four of the fluconazole-evolved strains (FLU-A, FLU-B, FLU-C, and FLU-A2) were also observed to have a small amount (2% to 4%) of lanosterol present, whereas this sterol was absent in the FLU-C2 strain.

**FIG 4:**
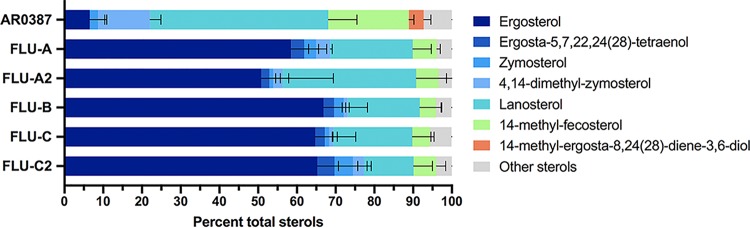
Sterol profiles of C. auris fluconazole-evolved strains grown in RPMI media supplemented with fluconazole reveal a lack of 14-methyl-ergosta-8,24(28)-dienol-3,6-diol. The major constituent sterols for AR0387 and fluconazole-evolved strains at the exponential-growth phase in RPMI media are shown as a proportion of total cellular sterols. Error bars for each data point represent the standard deviations of results from three independent measurements of technical replicates.

**FIG 5:**
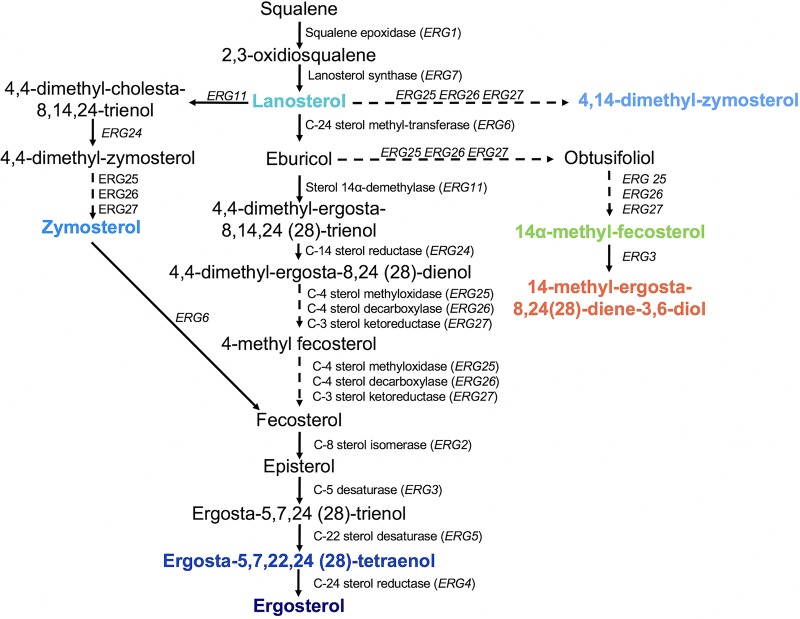
Predicted C. auris sterol biosynthesis pathway. The major constituent sterols identified in sterol profiles are shown with corresponding colors.

Following growth in RPMI media supplemented with fluconazole, the sterol profiles of each of the fluconazole-evolved strains were dramatically different from that of AR0387 (Fig. 4). While ergosterol was still the predominant sterol among all five fluconazole-evolved strains, lanosterol (46.0% ± 7.4%) and 14-methyl-fecosterol (21.3% ± 4.8%) were observed to be the two most prevalent sterols in AR0387. In Candida albicans, 14-methyl-fecosterol is a known substrate of the sterol-desaturase enzyme encoded by *CaERG3*, which catalyzes the conversion of 14-methyl-fecosterol to the toxic sterol associated with the antifungal activity of the triazoles, 14-methyl-ergosta-8,24(28)-dienol-3,6-diol (Fig. 5). Additionally, 14-methyl-ergosta-8,24(28)-dienol-3,6-diol comprised 3.8% ± 1.9% all sterols present in AR0387, while this sterol was absent in the sterol profiles of all fluconazole-evolved strains. As mutations in the sterol-desaturase-encoding gene *ERG3* have been observed to contribute to fluconazole resistance in other species of *Candida* and notable differences in the amounts of cellular 14-methyl-fecosterol and 14-methyl-ergosta-8,24(28)-dienol-3,6-diol were observed between AR0387 and the fluconazole-evolved strains, sequencing of the C. auris gene (B9J08_003737) with the highest degree of homology to C. albicans *CaERG3* was performed. However, no mutation in C. auris *ERG3* was observed in any of the fluconazole-evolved strains.

### Fluconazole-evolved strains exhibit significantly reduced fluconazole uptake.

**FIG 6:**
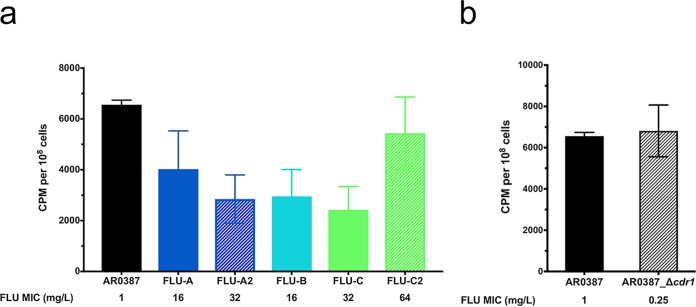
Decreased intracellular accumulation of [^3^H]fluconazole among fluconazole-evolved strains. [^3^H]-labeled fluconazole uptake was determined in (a) fluconazole-evolved strains and (b) a *CDR1* deletion strain and was compared to that shown by parental clinical isolate AR0387. FLU, fluconazole. Error bars for each data point represent standard deviations of results from six independent measurements of technical replicates. Intracellular accumulation of [^3^H]fluconazole was significantly lower in FLU-A, FLU-A2, FLU-B, and FLU-C than in AR0387 (*P* = 0.0021, *P* < 0.0001, *P* < 0.0001, and *P* < 0.0001, respectively), while accumulation in FLU-C2 did not significantly differ from that seen in AR0387 (*P* = 0.2842) and no difference in accumulation between AR0387 and AR0387_Δ*cdr1* was seen (*P* = 0.9976|).

As triazoles (including fluconazole) have previously been shown to enter the cells of C. albicans via facilitated diffusion, deficient drug importation was next examined as a potential mechanism contributing to the increased fluconazole resistance among the fluconazole-evolved strains (24). As previously described, the accumulation of [^3^H]fluconazole was assessed for AR0387 and each fluconazole-evolved strain, as well as for a previously characterized strain of AR0387 where the *CDR1* gene had been deleted (AR0387_Δ*cdr1*), following 2 h glucose starvation in YNB media without carbon source supplementation (20). [^3^H]fluconazole accumulation was observed to be reduced by approximately 50% in four of the fluconazole-evolved strains (FLU-A, FLU-B, FLU-C, and FLU-A2) relative to than that observed in AR0387, while accumulation in FLU-C2 did not significantly differ from that in AR0387 (5,438 versus 6,560 cpm, respectively; *P* = 0.2842) (Fig. 6a). Importantly, there was no difference in [^3^H]fluconazole accumulation between AR0387 and AR0387_Δ*cdr1* (6,560 and 6,813 cpm, respectively; *P* = 0.9976) (Fig. 6b), confirming that the conditions used in this study for glucose starvation were adequate to remove the activity of this known C. auris resistance effector.

### Mutations in *TAC1B* are associated with significantly increased expression of *CDR1*.

Gain-of-function (GOF) mutations in zinc-cluster transcription factor genes, such as C. albicans genes *CaUPC2*, *CaMRR1*, and *CaTAC1*, represent a well-characterized mechanism of fluconazole resistance among other species of *Candida* (15). To determine if similar mutations might have been contributing to the fluconazole resistance among the fluconazole-evolved strains in these studies, the C. auris genes with the highest degree of homology to C. albicans transcriptional regulatory genes *CaUPC2*, *CaMRR1*, and *CaTAC1*, here named *UPC2* (B9J08_000270), *MRR1* (B9J08_004061), *TAC1A* (B9J08_004819), and *TAC1B* (B9J08_004820), were identified by BLAST and gene orthology analysis and sequencing was performed. As two *C. auris* genes possessing very high degrees of homology with CaTAC1 were identified (TAC1A and TAC1B share 25.6% predicted peptide sequence identity with each other and share 30.6 and 26.9% identity with CaTAC1, respectively), both were included in this study. While no mutations were identified in *TAC1A* or *MRR1*, all five fluconazole-evolved strains were found to have mutations encoding amino acid substitutions in *TAC1B* (Table 1). Both the FLU-A strain and the corresponding second-generation derivative FLU-A2 were found to harbor a mutation encoding the amino acid substitution R495G, while the FLU-B, FLU-C, and FLU-C2 strains were found to possess a mutation encoding the amino acid substitution F214S. Neither of these mutations corresponds to previously characterized GOF mutations in *CaTAC1* or to orthologous genes from other species of *Candida*. However, these mutations are predicted to alter residues near or within the conserved fungal transcription factor middle homology region (MHR) of Tac1Bp, and multiple mutations encoding amino acid substitutions in the MHR of *CaTAC1* have previously been reported to be associated with fluconazole resistance (25). Additionally, a sole mutation in *UPC2* encoding the amino acid substitution M365I was identified in FLU-C2, and this mutation similarly alters a residue predicted to reside within the MHR of Upc2p.

**TABLE 1:** Sequencing of C. auris *MRR1*, *TAC1A*, *TAC1B*, and *UPC2* among fluconazole-evolved strains

Gene	Clinical isolate or strain					
	AR0387	FLU-A	FLU-A2	FLU-B	FLU-C	FLU-C2
*MRR1*	WT	WT	WT	WT	WT	WT
*TAC1A*	WT	WT	WT	WT	WT	WT
*TAC1B*	WT	R495G	R495G	F214S	F214S	F214S
*UPC2*	WT	WT	WT	WT	WT	M365I

**FIG 7:**
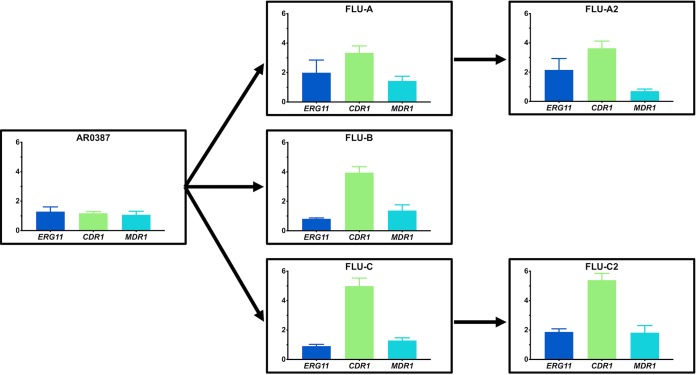
Significantly increased relative expression of C. auris *CDR1* among fluconazole-evolved strains grown to the exponential-growth phase in RPMI media. The levels of expression of C. auris *ERG11*, *CDR1*, and *MDR1* in AR0387 and the fluconazole-evolved strains were determined following culturing to the exponential-growth phase at 30°C in RPMI media. The expression level for each sample is shown relative to that of the respective gene in AR0387. Arrows between graphs indicate the lineage of each fluconazole-evolved strain from the parental AR0387. Error bars for each data point represent standard errors of the means of results from three biological replicates each performed with three technical replicates. The level of expression of C. auris *CDR1* among all fluconazole-evolved strains was observed to be significantly higher (3.3 to 5.4-fold) than that of AR0387 (*P* < 0.0001 for each individual comparison). Differences in the levels of expression of *CDR1* between the sequential evolved strains were not found to be significant (FLU-A versus FLU-A2, *P* = 0.7186; FLU-C versus FLU-C2, *P* = 0.3855).

In an effort to ascertain if the identified mutations in *TAC1B* and *UPC2* may be associated with altered expression of potential resistance effectors among the fluconazole-evolved strains, the relative expression levels of *ERG11*, *CDR1*, and *MDR1* were evaluated by reverse transcription-quantitative PCR (RT-qPCR). To accomplish this, AR0387 and each of the fluconazole-evolved strains were grown to the exponential-growth phase in RPMI media, and RNA was extracted as previously described. The expression of each gene of interest relative to AR0387 was assessed using the ΔΔ*C_T_* (threshold cycle) method and the C. auris *ACT1* housekeeping gene (B9J08_000486) (20). The level of expression of *CDR1* was found to be significantly (3-to-5-fold) higher in all five fluconazole-evolved strains than in AR0387 (*P* < 0.0001 for all evolved strains compared to AR0387) (Fig. 7). This level of *CDR1* expression is similar to that previously described among extensively fluconazole-resistant C. auris clinical isolates (20). Additionally, subtle variations in the levels of expression of the *ERG11* and *MDR1* genes, not exceeding 2.1-fold higher than the levels seen with AR0387, were also observed among individual fluconazole-evolved strains.

As copy number variations (CNVs) among genes encoding fluconazole resistance effectors, such as *ERG11*, have previously been reported among clinical isolates and laboratory strains of C. auris, qPCR amplifications from genomic DNA were performed to assay for CNV among the effectors *ERG11*, *CDR1*, and *MDR1*, as well as *TAC1B*, for each of the fluconazole-evolved strains. (26, 27). For each gene of interest, three primer sets spanning the open reading frame were utilized. While no alteration in the copy number of *ERG11*, *CDR1*, or *MDR1* was observed, the second-generation fluconazole-evolved FLU-A2 strain was found to show a 2-fold increase in the copy number of *TAC1B*, which was not evident in other evolved strains (see Fig. S1 in the supplemental material).

10.1128/mBio.00365-20.1 FIG S1 Copy number variation of *TAC1B* among fluconazole-evolved strains as determined by qPCR. Gene copy numbers of *TAC1B* across fluconazole-evolved strains and the parental AR0387 were determined by qPCR with three independent primer sets spanning the open reading frame and compared to the housekeeping gene *ACT1.* Error bars for each data point represent standard errors of the means of results determined for three biological replicates performed with three technical replicates. Each biological replicate was performed using an independent primer set spanning the open reading frame. Download FIG S1, TIF file, 2.6 MB. Copyright © 2020 Rybak et al. 2020 Rybak et al. This content is distributed under the terms of the Creative Commons Attribution 4.0 International license.

### *TAC1B* mutations identified during *in vitro* evolution studies are also present among fluconazole-resistant C. auris clinical isolates.

**FIG 8:**
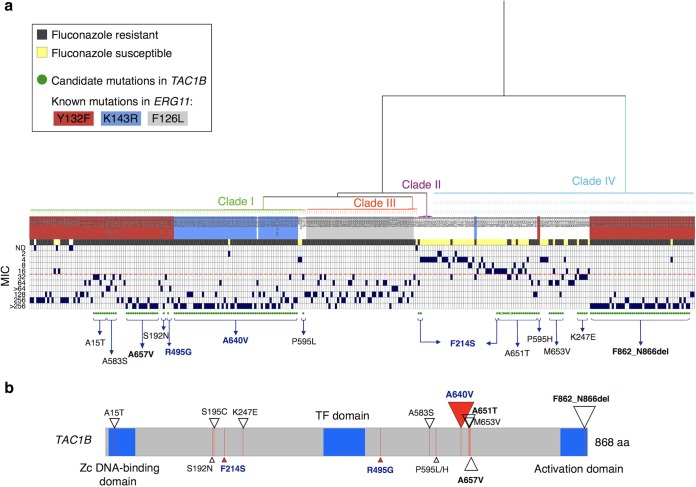
*TAC1B* point mutations and fluconazole susceptibility in C. auris. (a) Phylogenetic tree of SNPs identified from 304 C. auris whole-genome sequences from four major clades (I, II, III, and IV). Isolate label backgrounds are color coded for known mutations in *ERG11* (B9J08_001448) (Y132F, K143R, F126L). Susceptibility to fluconazole is depicted as resistant (dark gray) or susceptible (yellow), and the MIC values are indicated as dark-blue boxes. The red dotted line indicates the tentative fluconazole MIC breakpoint (≥32 mg/liter). Green circles indicate isolates harboring non-clade-specific nonsynonymous mutations or gain-of-function mutations in *TAC1B* (B9J08_004820), with filled circles corresponding to percent alternative allele of ≥0.8, while open green circles correspond to percent alternative allele of 0.67 to 0.79. The specific mutation is indicated for each isolate(s). Mutations in bold/dark blue arose in *in vitro* evolution experiments or were functionally tested in this study and found to be associated with increased resistance to fluconazole in C. auris. (b) Mutations and locations in *TAC1B* protein sequence associated with azole resistance are indicated using triangles. Mutations indicated with bold/dark blue (red triangles) arose in *in vitro* evolution experiments or were functionally tested in this study and associated with increased resistance to fluconazole in C. auris. The size of the triangle indicates the number of isolates from this study harboring the mutation (range, 1 to 57 isolates).

Interrogation of a data set consisting of whole-genome sequencing data for 304 globally distributed C. auris isolates representing each of the four major clades revealed 14 nonsynonymous *TAC1B* mutations and one deletion, excluding sites which are fixed in all isolates within a clade and which are present in both sensitive and resistant isolates (Fig. 8; see also Table S1 in the supplemental material) (27). In total, mutations in *TAC1B* were identified among 165 (54%) isolates. Additionally, 50% (148) of the isolates in this collection with available susceptibility data were found to be fluconazole resistant (MIC of ≥32mg/liter) and to possess a mutation in *TAC1B*. Furthermore, the two *TAC1B* mutations that arose during *in vitro* drug selection were found to be present among fluconazole-resistant clinical C. auris isolates, suggesting a possible role in clinical fluconazole resistance. R495G was found in a single clade I isolate, and the F214S change was found in 2 isolates from clade II and in 1 isolate from clade IV (Fig. 8; see also Table S1). Notably, a mutation encoding the A640V amino acid substitution was found to be the most common among clinical isolates, found in 57 clade I isolates from 7 countries and always present with the *ERG11* mutation encoding the K143R amino acid substitution. Nearly all (98.2%) of the isolates with A640V and K143R mutations displayed high-level fluconazole resistance (>64 mg/liter). Other common *TAC1B* mutations found included A657V in 15 clade I isolates and the frameshift mutation F862_N866del in 46 clade IV isolates. These mutations appeared in isolates with the *ERG11* Y132F variant, and these isolates were found to have markedly high MIC values (Fig. 8), suggesting that these mutations may provide additive fluconazole resistance effects. Comparison of Tac1B protein sequences indicated that C. auris A657V corresponds to the CaTac1 GOF mutation A736V associated with increased triazole resistance in C. albicans. Additionally, we observed three novel *TAC1B* mutations in clade IV isolates lacking resistance-associated mutations in *ERG11*, including K247E (*n* = 5), M653V (*n* = 7), and A651T (*n* = 16), six resistant isolates from clade I which harbored two *TAC1B* mutations (A15T and S195C), and two different mutations affecting the P595 site (P595L in clade I and P595H in clade IV).

10.1128/mBio.00365-20.2 TABLE S1 *TAC1A* and *TAC1B* mutations observed among the members of a global collection of C. auris isolates. Variants shown in bold are unique to resistant isolates. Variants shown in orange were identified through *in vitro* evolution experiments. Variants shown in yellow were found to directly contribute to fluconazole resistance. Variants shown in italics were uniquely found together. Percent values shown represent percentages of isolates with the indicated *TAC1B* mutation from the indicated clade which were found to have fluconazole MICs of ≥32mg/liter. Download Table S1, DOCX file, 0.02 MB. Copyright © 2020 Rybak et al. 2020 Rybak et al. This content is distributed under the terms of the Creative Commons Attribution 4.0 International license.

### Mutations in *TAC1B* contribute to fluconazole resistance.

**FIG 9:**
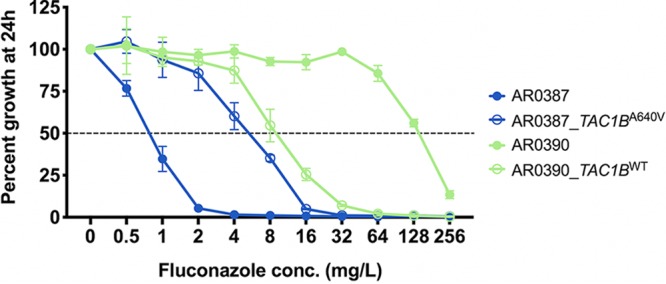
Fluconazole MIC for *TAC1B* strains. Percent growth of AR0387 and AR0390 and the corresponding derivative *TAC1B* strains with escalating concentrations of fluconazole was measured at 24 h. Percent growth was determined relative to the corresponding untreated controls as assessed by absorbance at OD_600_. Growth inhibition of 50% relative to the untreated control is shown as a dotted horizontal line. Error bars for each data point represent the standard deviations of results from three independent measurements of technical replicates.

As mutations in *TAC1B* were identified in a large proportion of fluconazole-resistant C. auris clinical isolates and as the mutation encoding the amino acid substitution A640V was found to be the most prevalent among the members of this large collection of clinical isolates, the direct impact of this mutation on fluconazole susceptibility was next determined using a Cas9-mediated transformation system. To accomplish this, the *TAC1B* allele from the previously characterized fluconazole-resistant C. auris AR0390 clinical isolate (also known as B11205, an isolate from clade I), which contains the mutation encoding the amino acid substitution A640V, was introduced into the fluconazole-susceptible AR0387 clinical isolate by the use of Cas9 ribonucleoproteins (Cas9-RNP) and the *SAT-FLP* system as previously described (20). Two independent positive-testing transformants were obtained, and the fluconazole MICs were determined by broth microdilution. Introduction of the *TAC1B*^A640V^ allele into the native *TAC1B* locus was observed to increase the fluconazole MIC 8-fold relative to the parental AR0387 (Fig. 9). Conversely, when the same methods were used to introduce the wild-type *TAC1B* allele to isolate AR0390 (which harbors the *TAC1B* mutation encoding A640V), a 16-fold decrease in fluconazole MIC was observed (Fig. 9). The fluconazole MICs did not differ between independent transformants.

## DISCUSSION

C. auris has rapidly become a fungal pathogen of global concern. Among the characteristics most notably distinguishing this organism from other species of *Candida*, the prevalence of fluconazole resistance is of clear clinical concern as fluconazole remains the most commonly prescribed antifungal worldwide. While mutations in the *ERG11* gene are strongly associated with clinical fluconazole resistance in C. auris, the presence of this mechanism alone poorly explains the entirety of resistance observed clinically, and the roles of other genetic and molecular mechanisms contributing to fluconazole resistance in this organism remain largely unknown.

To date, the predominance of knowledge of the molecular mechanisms of fluconazole resistance among species of *Candida* comes from experience studying C. albicans. In this organism, the most commonly reported mechanisms of fluconazole resistance include mutations in the gene encoding the target of the triazole antifungals, *CaERG11*, and overexpression of either *CaERG11* or genes encoding multidrug-efflux pumps such as *CaCDR1*, *CaCDR2*, and *CaMDR1* (12, 15, 28). In fact, among a collection of 63 unrelated fluconazole-resistant C. albicans clinical isolates, all were found to exhibit one of these resistance mechanisms and the vast majority possessed a combination of mechanisms. Specifically, 87% were found to have missense mutations in *CaERG11*, 75% exhibited elevated expression of *CaERG11*, 77% exhibited elevated expression of *CaCDR1* and *CaCDR2*, and 21% exhibited elevated expression of *CaMDR1* to levels known to contribute to fluconazole-resistance (12, 28). The increased expression of C. albicans fluconazole resistance effectors has been extensively studied and in the majority of isolates is directly attributable to GOF mutations in zinc-cluster transcription factor genes such as *CaUPC2*, *CaTAC1*, and *CaMRR1* (contributing to the increased expression of *CaERG11*, both *CaCDR1* and *CaCDR2*, and *CaMDR1*, respectively) (15, 28–30). Other C. albicans mechanisms of fluconazole resistance, such as loss-of-function (LOF) mutations in *CaERG3*, have also been reported but are much less commonly identified among clinical isolates (31).

In this work, we utilized *in vitro* evolution to create a collection of isogenic C. auris strains with elevated fluconazole MICs in an effort to identify novel genetic determinants of fluconazole resistance. Strains with significant (8-fold to 32-fold) increases in fluconazole MICs were obtained after a total of only 96 h of growth in fluconazole-supplemented media, and this resistance was observed to be stable after subsequent culturing on fluconazole-free media. This rapid emergence of increased fluconazole resistance is particularly concerning, considering that patients being treated for infections caused by C. auris have acquired antifungal-resistant infections while receiving therapy and that the fluconazole-resistant strains in these experiments were obtained after only a single passage in media supplemented with fluconazole at clinically relevant concentrations (8 to 32 mg/liter) (23). The rapidity of this emergence of fluconazole resistance may be influenced by the haploid nature of the C. auris genome, where acquisition of even a recessive mutation may make a larger immediate contribution to resistance than has been found to occur with other diploid species of *Candida* such as C. albicans, where a loss-of-heterozygosity event may be required to observe the maximal impact of a single resistance mutation (32). Furthermore, a notable proportion of C. auris clinical isolates have been observed to exhibit increased copy numbers of resistance-associated genes, as was identified in this study with the increased *TAC1B* copy numbers seen in the second-generation fluconazole-evolved FLU-A2 strain (which concomitantly harbored a R495G-encoding mutation in *TAC1B*) (27).

Surprisingly, no mutations in C. auris *ERG11* were identified among the fluconazole-evolved strains created in this study, and all five strains were found to harbor one of two mutations in *TAC1B* (encoding F214S and R495G), a gene with a high degree of homology to the well-characterized C. albicans transcriptional regulator gene *CaTAC1.* Intriguingly, two of the three first-generation evolved strains, FLU-B and FLU-C, were found to have acquired the same *TAC1B* mutation (F214S) even after selection following exposure to different concentrations of fluconazole (8 and 32 mg/liter, respectively). Furthermore, RT-qPCR revealed that each of the five *TAC1B* mutant strains exhibited elevated expression of the ABC-type efflux pump-encoding gene *CDR1*, as has been observed with clinical C. albicans isolates possessing a GOF mutation in *CaTAC1* (29). Importantly, the degree of *CDR1* overexpression observed among fluconazole-evolved strains was similar to that previously reported among fluconazole-resistant clinical C. auris isolates (20).

The clinical relevance of mutations in C. auris *TAC1B* was further corroborated upon large-scale analysis of whole-genome sequencing data for over 300 C. auris clinical isolates. Among the members of this global collection, the majority (54%) of the isolates were found to have mutations in *TAC1B*, and 90% of all isolates with *TAC1B* mutations were resistant to fluconazole. A total of 14 nonsynonymous *TAC1B* mutations and one deletion were identified, and this included both of the mutations identified in fluconazole-evolved strains, as well as a single mutation (encoding A640V) which was found among 57 clinical isolates of C. auris from clade I. Subsequently, Cas9-RNP-mediated genetic manipulations demonstrated that the mutation in *TAC1B* encoding the A640V amino acid substitution, the most common mutation found among fluconazole-resistant clinical isolates of C. auris, was sufficient alone to elevate fluconazole resistance by 8-fold. Thus, these data definitively demonstrate that mutations in C. auris *TAC1B* represent a novel genetic determinant of clinical fluconazole resistance.

Additional characterization of the fluconazole-evolved C. auris strains created in this study was also performed to identify potential changes in cellular sterol composition and fluconazole uptake which might also be associated with increased fluconazole resistance in C. auris. Previously, analysis of sterol profiles had revealed significant changes in cellular sterol composition among fluconazole-resistant clinical isolates of *Candida* possessing mutations in ergosterol biosynthesis genes. In one example, a clinical C. albicans isolate with pan-triazole resistance and harboring mutations in both *CaERG11* and *CaERG5* was found to produce no detectable ergosterol while instead accumulating a large amount of ergosta-5,7-dienol (82% of total sterols) (33). More-subtle sterol profile changes were also reported in a multidrug-resistant clinical isolate of C. parapsilosis possessing a loss-of-function mutation in *CpERG3* (encoding G111R). This isolate was found to accumulate large amounts of ergosta-7,22-dienol (72% of total sterols), a substrate of *CpERG3* not typically present in such large proportions (22).

Thus, to assay for changes in ergosterol biosynthesis which might be associated with the fluconazole resistance phenotype, comprehensive sterol profiling of the fluconazole-evolved C. auris strains was undertaken. Following growth in RPMI media without fluconazole supplementation, the only notable difference in levels of sterols among the fluconazole-evolved strains and AR0387 was a lack of detectable lanosterol, the substrate of *ERG11*, in the second-generation evolved FLU-C2 strain. It is tempting to speculate that this lack of lanosterol may be associated with the mutation in *UPC2* which is unique to this fluconazole-evolved strain, as GOF mutations in *CaUPC2* have been shown to increase expression of ergosterol biosynthesis genes, including *CaERG11* (28). However, further interrogation of the C. auris *UPC2* regulon and this potential resistance-associated mutation is clearly needed to definitively associate this phenotype with the observed mutation in C. auris *UPC2*.

Following growth in RPMI media supplemented with 16 mg/liter (a concentration representative of the plasma concentration of fluconazole in patients being treated for candidemia), a stark difference in the sterol profiles of AR0387 and all fluconazole-evolved strains was observed. Whereas ergosterol remained the principal sterol present in all fluconazole-evolved strains (comprising more than 50% of sterols), lanosterol predominated the total cellular sterols in the parental AR0387 isolate (46%). Additionally, 14-methyl-ergosta-8,24(28)-diene-3,6-diol, a sterol proposed to be toxic and important for the antifungal activity of the triazoles, was uniquely found in AR0387 (4% of total sterols) in conjunction with a large amount of 14-methyl-fecosterol (21%), the direct precursor of this speculated toxic sterol (34). However, this change in the sterol profile of AR0387 under conditions of fluconazole treatment is consistent with previously reported changes in fungal sterols with suprainhibitory concentrations of fluconazole (33, 35). Therefore, these changes in the sterol profile of AR0387 may be related to the lower fluconazole MIC for this clinical isolate (1 mg/liter) whereas the maintained ergosterol content observed in fluconazole-evolved strains may be a direct consequence of the identified mutations in C. auris *TAC1B* or a result of the decreased fluconazole sensitivity of these fluconazole-evolved strains. Further investigation of the *TAC1B* regulon and the impact of *TAC1B* mutations on ergosterol biosynthesis and sterol profiles is required.

While the mechanism of triazole uptake in fungi has remained unknown to date and decreased drug uptake is not a mechanism which has been demonstrated to contribute to fluconazole resistance among clinical isolates of *Candida*, it is has been suggested that clinical isolates of *Candida* with reduced fluconazole sensitivity may exhibit altered fluconazole uptake (24). To interrogate the potential role of altered fluconazole uptake in the resistance observed among the fluconazole-evolved strains created in this study, analysis of [^3^H]fluconazole uptake was performed. Among the fluconazole-evolved strains, this analysis revealed an intriguing decrease in [^3^H]fluconazole uptake in 4 of the 5 strains compared to AR0387. All strains except for FLU-C2 were found to accumulate approximately 50% less [^3^H]fluconazole under energy-depleted conditions. While the reason for the higher degree of [^3^H]fluconazole uptake in the FLU-C2 strain remains unknown, again it is tempting to speculate whether the mutation in *UPC2* which is unique to this strain may be involved, particularly as it contrasts with the diminished uptake in the related first-generation evolved FLU-C strain. Further investigation of the role of altered fluconazole uptake in resistance among clinical isolates of C. auris and the impact of mutations in either *TAC1B* or *UPC2* on fluconazole uptake is clearly merited.

While the findings from the experiments described here are intriguing and of clear clinical significance, this study also had limitations. The *in vitro* evolution experiments described were performed in a single genetic background and using only fluconazole. Thus, it cannot be determined if different mechanisms of triazole resistance would be observed if similar studies were conducted with different clinical isolates of C. auris or different agents of the triazole class of antifungals. Additionally, while similarities between the fluconazole-evolved strains in this work and clinical isolates of *Candida* possessing known GOF mutations in close homologs of *TAC1B* are apparent, such as the increased expression of *CDR1*, further characterization of the C. auris *TAC1B* regulon is needed to fully understand the potential similarities and differences between the mutations in *TAC1B* and known mechanisms of fluconazole resistance in other species of *Candida.* That withstanding, taken together, the findings of these studies serve to demonstrate that mutations in *TAC1B* both represent a potent genetic determinant contributing to clinical fluconazole resistance in C. auris and are prevalent among the members of a large global collection of fluconazole-resistant clinical isolates. Further studies characterizing the interplay between mutations in *ERG11* and *TAC1B* and the delineation of the *TAC1B* regulon in C. auris are needed.

## MATERIALS AND METHODS

### Isolate, strains, and growth media used in this study.

Clinical isolates AR0387 and AR0390 were made available by the CDC and FDA AR Isolate Bank as part of the C. auris collection of isolates. All constructed strains and clinical isolates were grown in YPD liquid media (1% yeast extract, 2% peptone, and 2% dextrose) at 30°C in a shaking incubator unless otherwise indicated. Frozen stocks of all strains and clinical isolates were prepared with 50% sterile glycerol and were maintained at –80°C.

### MIC determination.

Fluconazole (Sigma) was prepared in dimethyl sulfoxide (DMSO). As previously described, a modified version of the Clinical and Laboratory Standards Institute document M27 methodology utilizing broth microdilution and RPMI liquid media and reading absorbance at 600 nm on a BioTek Synergy 2 microplate reader (BioTek, Winooski, VT) was used to determine the fluconazole MIC as the lowest concentration at which 50% inhibition of growth was obtained (36). All susceptibility testing was performed in technical triplicate and biological duplicate.

### Comprehensive sterol profiling.

Fluconazole-evolved strains and the parental clinical isolate were grown to the exponential-growth phase at 30°C in RPMI liquid media with or without fluconazole supplemented at 16 mg/liter. Alcoholic KOH was used to extract nonsaponifiable lipids. A vacuum centrifuge (Heto) was used to dry samples, which were then derivatized by adding 100 μl 90% N,O-bis(trimethylsilyl)-trifluoroacetamide–10% tetramethylsilane (TMS) (Sigma) and 200 μl anhydrous pyridine (Sigma) while heating at 80°C for 2 h as previously described (22, 34). gas chromatography-mass spectroscopy (GC-MS) (with a Thermo 1300 gas chromatography system coupled to a Thermo ISQ mass spectrometer; Thermo Scientific) was used to analyze and identify TMS-derivatized sterols through comparison of the retention times and fragmentation spectra for known standards. Sterol profiles for each sample were determined by analyzing the integrated peak areas from GC-MS data files using Xcalibur software (Thermo Scientific). All sterol analysis was performed in biological triplicate. Error bars for each data point represent the standard deviations of results from three independent measurements of technical replicates.

### Assessment of [^3^H]fluconazole uptake.

C. auris isolates and fluconazole-evolved strains were subjected to glucose starvation for 3 h, and 200-μl volumes of concentrated cell pellets were added to 250 μl of YNB without glucose and 50 μl of freshly diluted 0.77 μM [^3^H]fluconazole, yielding a final [^3^H]fluconazole concentration significantly below the MIC of each strain or isolate being tested (23.6 pg/liter). Samples were then incubated at 30°C for 24 h, after which 200 μl of each sample was transferred to 5 ml of stop solution (YNB plus 20 mM [6 mg/liter] unlabeled fluconazole) in a 14-ml round-bottom tube. Samples were then filtered and dried on glass fiber filters and then washed with another 5 ml of stop solution, and the filters and cells were then transferred to a 5-ml scintillation vial. A Beckman Coulter scintillation analyzer was then used to quantify the radioactivity of each filter following the addition of 3 ml of scintillation cocktail (Ecoscint XR, National Diagnostics). Experiments were performed with six biological replicates, and all results were normalized to cpm per 1 × 10^8^ cells. Statistical comparisons were made using a one-way analysis of variance (ANOVA) followed by a Tukey test, and the *P* values presented represent adjusted values.

### Assessment of copy number variation by qPCR and relative gene expression by reverse transcription-quantitative PCR.

For assessment of gene copy number variation, genomic DNA was isolated from each isolate or strain, and qPCR was performed by the use of three independent primer sets spanning the open reading frame of each gene of interest and the housekeeping gene *ACT1*, using SYBR green per the manufacturer’s instructions and as previously described (26). For assessment of relative gene expression levels, C. auris isolates and strains were inoculated into 2 ml of RPMI broth buffered with morpholinepropanesulfonic acid (MOPS) to pH 7.0 and grown overnight at 30°C for initiation. Overnight cultures were then diluted to an optical density at 600 nm (OD_600_) of 0.1 in 10 ml of RPMI media with or without 16 mg/ml of fluconazole and placed in a 50-ml conical tube. Cultures were then incubated for 10 h and then confirmed to be in the exponential-growth phase under these conditions, after which the cells were collected by centrifugation, with storage of the cell pellets at –80°C until isolation of RNA was performed. Synthesis of cDNA was performed using a RevertAid RT kit (Thermo Scientific) per the manufacturer’s instructions. C. auris *ACT1*, *ERG11*, *CDR1*, and *MDR1* were then amplified from cDNA using SYBR green, PCR master mix, and previously described parameters (20). All experiments were performed in biological and technical triplicate. The 2^−ΔΔ^*^CT^* method was used to calculate the relative levels of expression of each gene of interest, and standard errors were determined using Δ*C_T_* values as previously described (37, 38). Error bars for each data point represent standard errors of the means of results from three biological replicates performed with three technical replicates. Statistical comparisons were made using a one-way ANOVA followed by a Tukey test, and the *P* values presented represent adjusted values. Primers are listed in Table S2 in the supplemental material.

10.1128/mBio.00365-20.3 TABLE S2 Oligonucleotides used in this study. Download Table S2, DOCX file, 0.02 MB. Copyright © 2020 Rybak et al. 2020 Rybak et al. This content is distributed under the terms of the Creative Commons Attribution 4.0 International license.

### Variant identification.

*TAC1A* (B9J08_004819) and *TAC1B* (B9J08_004820) mutations were identified in a set of 304 globally distributed Candida auris isolates representing clades I, II, III, and IV (27). For this data set, analysis of read quality and filtering was performed using FastQC v0.11.5 and PRINSEQ v0.20.3 (39) with “-trim_left 15 -trim_qual_left 20 -trim_qual_right 20 -min_len 100 -min_qual_mean 25 -derep 14.” Then, paired-end reads were aligned to C. auris assembly strain B8441 (GenBank accession no. PEKT00000000.2 [18]) using BWA mem v0.7.12 (40), and variants were identified using GATK v3.7 (41) with the haploid mode and GATK tools (RealignerTargetCreator, IndelRealigner, HaplotypeCaller for both single nucleotide polymorphisms [SNPs] and indels, CombineGVCFs, GenotypeGVCFs, GatherVCFs, SelectVariants, and Variant Filtration). Sites were filtered with Variant Filtration using “QD < 2.0 ǁ FS > 60.0 ǁ MQ < 40.0.” Genotypes were filtered if the minimum genotype quality value was <50, the percent alternative allele value was <0.8, or the depth value was <10 (https://github.com/broadinstitute/broad-fungalgroup/blob/master/scripts/SNPs/filterGatkGenotypes.py). Genomic variants were annotated and the functional effect predicted using SnpEff v4.3T (42). The annotated VCF file was used to determine the genotype of known mutation sites in *ERG11* (B9J08_001448) and mutations in *TAC1A* (B9J08_004819) and *TAC1B* (B9J08_004820).

### Antifungal susceptibility testing for global collection of isolates.

Fluconazole susceptibility testing was included for 294 of the 304 isolates included in whole-genome analyses. A total of 270 isolates were tested at the CDC as outlined by Clinical and Laboratory Standards Institute guidelines. Briefly, custom prepared microdilution plates (Trek Diagnostics, Oakwood Village, OH, USA) were used for fluconazole. Resistance to fluconazole was set at ≥32 mg/liter. This interpretive breakpoint was defined based on a combination of these breakpoints with those established for other closely related *Candida* species, epidemiological cutoff values, and the biphasic distribution of MICs between the isolates with and without known mutations for antifungal resistance (https://www.cdc.gov/fungal/candida-auris/c-auris-antifungal.html).

### Cas9-ribonucleoprotein-mediated transformations.

C. auris Cas9 and electroporation-mediated transformations were performed as previously described (20) with minor modification. The C. auris *TAC1B* alleles from AR0387 (*TAC1B*^WT^) and AR0390 (*TAC1B*^A640V^) were amplified from genomic DNA and then cloned into plasmid pBSS2 using restriction enzymes SacII and EagI, yielding plasmids pBSS2-*TAC1B*^WT^ and pBSS2-*TAC1B*^A640V^. Repair templates for each allele of interest were then amplified from each plasmid using primers that also introduced approximately 50 bases of homology targeting the *TAC1B* loci to the 3′ end of the repair templates. Primers are listed in Table S2. Electrocompetent C. auris cells were prepared as previously described. Approximately 4 μM concentrations of dual Cas9-RNP constructs targeting both the *TAC1B* allele and the sequence immediately downstream of the open reading frame and 1 μg of repair template were mixed with cells prior to electroporation performed according to the C. albicans protocol on a GenePulsar Xcell (Bio-Rad) (24). Cells were then allowed to recover for 4 to 6 h in YPD with incubation in a shaking incubator at 30°C. Transformants were then selected by plating recovered cells on YPD plates supplemented with 400 μg/ml of nourseothricin. Integration of the repair template at the targeted loci was then confirmed by PCR for all transformants. The *FLP* recombinase was then induced by growing positive-testing transformants in YPM (1% yeast extract, 2% peptone, and 2% maltose) to mediate loss of the *SAT1-FLP* cassette. All final strains that were identified as having lost the *SAT1-FLP* cassette by replica plating as previously described were then again confirmed by sequencing (18).

### Data availability.

All data from Illumina sequences analyzed in this project are available in the NCBI SRA under BioProject accession no. PRJNA328792, PRJNA470683, and PRJNA493622. A set of isolates are available from the CDC and FDA Antimicrobial Resistance (AR) Isolate Bank (https://www.cdc.gov/drugresistance/resistance-bank/index.html).

## References

[1] Satoh K, Makimura K, Hasumi Y, Nishiyama Y, Uchida K, Yamaguchi H 2009 Candida auris sp. nov., a novel ascomycetous yeast isolated from the external ear canal of an inpatient in a Japanese hospital. Microbiol Immunol 53:41–44. doi:10.1111/j.1348-0421.2008.00083.x. 19161556

[2] Lockhart SR, Etienne KA, Vallabhaneni S, Farooqi J, Chowdhary A, Govender NP, Colombo AL, Calvo B, Cuomo CA, Desjardins CA, Berkow EL, Castanheira M, Magobo RE, Jabeen K, Asghar RJ, Meis JF, Jackson B, Chiller T, Litvintseva AP 2017 Simultaneous emergence of multidrug-resistant Candida auris on 3 continents confirmed by whole-genome sequencing and epidemiological analyses. Clin Infect Dis 64:134–140. doi:10.1093/cid/ciw691. 27988485 PMC5215215

[3] Anonymous. 2019 Tracking *Candida auris*. https://www.cdc.gov/fungal/candida-auris/tracking-c-auris.html. Accessed 16 January 2020.

[4] Lockhart SR 2019 Candida auris and multidrug resistance: defining the new normal. Fungal Genet Biol 131:103243. doi:10.1016/j.fgb.2019.103243. 31228646 PMC12012538

[5] Caceres DH, Forsberg K, Welsh RM, Sexton DJ, Lockhart SR, Jackson BR, Chiller T 28 11 2019, posting date Candida auris: a review of recommendations for detection and control in healthcare settings. J Fungi (Basel) doi:10.3390/jof5040111. PMC6958335 31795175

[6] Anonymous. 2020 *Candida auris*: antifungal susceptibility testing and interpretation. Centers for Disease Control and Prevention, Atlanta, GA https://www.cdc.gov/fungal/candida-auris/c-auris-antifungal.html. Accessed January 16th.

[7] Ostrowsky B, Greenko J, Adams E, Quinn M, O’Brien B, Chaturvedi V, Berkow E, Vallabhaneni S, Forsberg K, Chaturvedi S, Lutterloh E, Blog D, C. auris Investigation Work Group. 2020 Candida auris isolates resistant to three classes of antifungal medications - New York, 2019. MMWR Morb Mortal Wkly Rep 69:6–9. doi:10.15585/mmwr.mm6901a2. 31917780 PMC6973342

[8] Chowdhary A, Prakash A, Sharma C, Kordalewska M, Kumar A, Sarma S, Tarai B, Singh A, Upadhyaya G, Upadhyay S, Yadav P, Singh PK, Khillan V, Sachdeva N, Perlin DS, Meis JF 2018 A multicentre study of antifungal susceptibility patterns among 350 Candida auris isolates (2009–17) in India: role of the ERG11 and FKS1 genes in azole and echinocandin resistance. J Antimicrob Chemother 73:891–899. doi:10.1093/jac/dkx480. 29325167

[9] Vallabhaneni S, Baggs J, Tsay S, Srinivasan AR, Jernigan JA, Jackson BR 2018 Trends in antifungal use in US hospitals, 2006–12. J Antimicrob Chemother 73:2867–2875. doi:10.1093/jac/dky270. 30295769

[10] Adam RD, Revathi G, Okinda N, Fontaine M, Shah J, Kagotho E, Castanheira M, Pfaller MA, Maina D 2019 Analysis of Candida auris fungemia at a single facility in Kenya. Int J Infect Dis 85:182–187. doi:10.1016/j.ijid.2019.06.001. 31185293

[11] Al Maani A, Paul H, Al-Rashdi A, Wahaibi AA, Al-Jardani A, Al Abri AMA, AlBalushi MAH, Al-Abri S, Al Reesi M, Al Maqbali A, Al Kasaby NM, de Groot T, Meis JF, Al-Hatmi A 23 10 2019, posting date Ongoing challenges with healthcare-associated Candida auris outbreaks in Oman. J Fungi (Basel) doi:10.3390/jof5040101. PMC6958405 31652825

[12] Flowers SA, Colon B, Whaley SG, Schuler MA, Rogers PD 2015 Contribution of clinically derived mutations in ERG11 to azole resistance in Candida albicans. Antimicrob Agents Chemother 59:450–460. doi:10.1128/AAC.03470-14. 25385095 PMC4291385

[13] Healey KR, Kordalewska M, Jimenez Ortigosa C, Singh A, Berrio I, Chowdhary A, Perlin DS 24 9 2018, posting date Limited ERG11 mutations identified in isolates of Candida auris directly contribute to reduced azole susceptibility. Antimicrob Agents Chemother doi:10.1128/AAC.01427-18. PMC6153782 30082281

[14] Escandon P, Chow NA, Caceres DH, Gade L, Berkow EL, Armstrong P, Rivera S, Misas E, Duarte C, Moulton-Meissner H, Welsh RM, Parra C, Pescador LA, Villalobos N, Salcedo S, Berrio I, Varon C, Espinosa-Bode A, Lockhart SR, Jackson BR, Litvintseva AP, Beltran M, Chiller TM 2019 Molecular epidemiology of Candida auris in Colombia reveals a highly related, countrywide colonization with regional patterns in amphotericin B resistance. Clin Infect Dis 68:15–21. doi:10.1093/cid/ciy411. 29788045

[15] Whaley SG, Berkow EL, Rybak JM, Nishimoto AT, Barker KS, Rogers PD 2016 Azole antifungal resistance in Candida albicans and emerging non-albicans Candida species. Front Microbiol 7:2173. doi:10.3389/fmicb.2016.02173. 28127295 PMC5226953

[16] Whaley SG, Zhang Q, Caudle KE, Rogers PD 24 11 2018, posting date Relative contribution of the ABC transporters Cdr1, Pdh1, and Snq2 to azole resistance in Candida glabrata. Antimicrob Agents Chemother doi:10.1128/AAC.01070-18. PMC6153852 30038038

[17] Ben-Ami R, Berman J, Novikov A, Bash E, Shachor-Meyouhas Y, Zakin S, Maor Y, Tarabia J, Schechner V, Adler A, Finn T 13 1 2017, posting date Multidrug-resistant Candida haemulonii and C. auris, Tel Aviv, Israel. Emerg Infect Dis doi:10.3201/eid2302.161486. PMC5324804 28098529

[18] Munoz JF, Gade L, Chow NA, Loparev VN, Juieng P, Berkow EL, Farrer RA, Litvintseva AP, Cuomo CA 2018 Genomic insights into multidrug-resistance, mating and virulence in Candida auris and related emerging species. Nat Commun 9:5346. doi:10.1038/s41467-018-07779-6. 30559369 PMC6297351

[19] Wasi M, Khandelwal NK, Moorhouse AJ, Nair R, Vishwakarma P, Bravo Ruiz G, Ross ZK, Lorenz A, Rudramurthy SM, Chakrabarti A, Lynn AM, Mondal AK, Gow NAR, Prasad R 2019 ABC transporter genes show upregulated expression in drug-resistant clinical isolates of Candida auris: a genome-wide characterization of ATP-binding cassette (ABC) transporter genes. Front Microbiol 10:1445. doi:10.3389/fmicb.2019.01445. 31379756 PMC6647914

[20] Rybak JM, Doorley LA, Nishimoto AT, Barker KS, Palmer GE, Rogers PD 27 3 2019, posting date Abrogation of triazole resistance upon deletion of CDR1 in a clinical isolate of Candida auris. Antimicrob Agents Chemother doi:10.1128/AAC.00057-19. PMC6437491 30718246

[21] Kim SH, Iyer KR, Pardeshi L, Munoz JF, Robbins N, Cuomo CA, Wong KH, Cowen LE 2019 Genetic analysis of Candida auris implicates Hsp90 in morphogenesis and azole tolerance and Cdr1 in azole resistance. mBio 10:e00346-19. doi:10.1128/mBio.00346-19. 30696744 PMC6355988

[22] Rybak JM, Dickens CM, Parker JE, Caudle KE, Manigaba K, Whaley SG, Nishimoto AT, Luna-Tapia A, Roy S, Zhang Q, Barker KS, Palmer GE, Sutter TR, Homayouni R, Wiederhold NP, Kelly SL, Rogers PD 24 8 2017, posting date Loss of C-5 sterol desaturase activity results in increased resistance to azole and echinocandin antifungals in a clinical isolate of Candida parapsilosis. Antimicrob Agents Chemother doi:10.1128/AAC.00651-17. PMC5571332 28630186

[23] Thaler F, Bernard B, Tod M, Jedynak CP, Petitjean O, Derome P, Loirat P 1995 Fluconazole penetration in cerebral parenchyma in humans at steady state. Antimicrob Agents Chemother 39:1154–1156. doi:10.1128/aac.39.5.1154. 7625804 PMC162699

[24] Mansfield BE, Oltean HN, Oliver BG, Hoot SJ, Leyde SE, Hedstrom L, White TC 2010 Azole drugs are imported by facilitated diffusion in Candida albicans and other pathogenic fungi. PLoS Pathog 6:e1001126. doi:10.1371/journal.ppat.1001126. 20941354 PMC2947996

[25] Nishimoto AT, Sharma C, Rogers PD 1 2 2019, posting date Molecular and genetic basis of azole antifungal resistance in the opportunistic pathogenic fungus Candida albicans. J Antimicrob Chemother doi:10.1093/jac/dkz400. PMC8204710 31603213

[26] Bhattacharya S, Holowka T, Orner EP, Fries BC 2019 Gene duplication associated with increased fluconazole tolerance in Candida auris cells of advanced generational age. Sci Rep 9:5052. doi:10.1038/s41598-019-41513-6. 30911079 PMC6434143

[27] Chow NA, Muñoz JF, Gade L, Berkow E, Li X, Welsh RM, Forsberg K, Lockhart SR, Adam R, Alanio A, Alastruey-Izquierdo A, Althawadi S, Belén Araúz A, Ben-Ami R, Bharat A, Calvo B, Desnos-Ollivier M, Escandón P, Gardam D, Gunturu R, Heath CH, Kurzai O, Martin R, Litvintseva AP, Cuomo CA 2020 Tracing the evolutionary history and global expansion of Candida auris using population genomic analyses. bioRxiv doi:10.1101/2020.01.06.896548. PMC7188998 32345637

[28] Flowers SA, Barker KS, Berkow EL, Toner G, Chadwick SG, Gygax SE, Morschhauser J, Rogers PD 2012 Gain-of-function mutations in UPC2 are a frequent cause of ERG11 upregulation in azole-resistant clinical isolates of Candida albicans. Eukaryot Cell 11:1289–1299. doi:10.1128/EC.00215-12. 22923048 PMC3485914

[29] Coste AT, Karababa M, Ischer F, Bille J, Sanglard D 2004 TAC1, transcriptional activator of CDR genes, is a new transcription factor involved in the regulation of Candida albicans ABC transporters CDR1 and CDR2. Eukaryot Cell 3:1639–1652. doi:10.1128/EC.3.6.1639-1652.2004. 15590837 PMC539021

[30] Dunkel N, Blass J, Rogers PD, Morschhauser J 2008 Mutations in the multi-drug resistance regulator MRR1, followed by loss of heterozygosity, are the main cause of MDR1 overexpression in fluconazole-resistant Candida albicans strains. Mol Microbiol 69:827–840. doi:10.1111/j.1365-2958.2008.06309.x. 18577180 PMC2678921

[31] Martel CM, Parker JE, Bader O, Weig M, Gross U, Warrilow AG, Rolley N, Kelly DE, Kelly SL 2010 Identification and characterization of four azole-resistant erg3 mutants of Candida albicans. Antimicrob Agents Chemother 54:4527–4533. doi:10.1128/AAC.00348-10. 20733039 PMC2976150

[32] Ford CB, Funt JM, Abbey D, Issi L, Guiducci C, Martinez DA, Delorey T, Li BY, White TC, Cuomo C, Rao RP, Berman J, Thompson DA, Regev A 2015 The evolution of drug resistance in clinical isolates of Candida albicans. Elife 4:e00662. doi:10.7554/eLife.00662. 25646566 PMC4383195

[33] Martel CM, Parker JE, Bader O, Weig M, Gross U, Warrilow AG, Kelly DE, Kelly SL 2010 A clinical isolate of Candida albicans with mutations in ERG11 (encoding sterol 14alpha-demethylase) and ERG5 (encoding C22 desaturase) is cross resistant to azoles and amphotericin B. Antimicrob Agents Chemother 54:3578–3583. doi:10.1128/AAC.00303-10. 20547793 PMC2934972

[34] Kelly SL, Lamb DC, Kelly DE, Manning NJ, Loeffler J, Hebart H, Schumacher U, Einsele H 1997 Resistance to fluconazole and cross-resistance to amphotericin B in Candida albicans from AIDS patients caused by defective sterol delta5,6-desaturation. FEBS Lett 400:80–82. doi:10.1016/s0014-5793(96)01360-9. 9000517

[35] Parker JE, Merkamm M, Manning NJ, Pompon D, Kelly SL, Kelly DE 2008 Differential azole antifungal efficacies contrasted using a Saccharomyces cerevisiae strain humanized for sterol 14 alpha-demethylase at the homologous locus. Antimicrob Agents Chemother 52:3597–3603. doi:10.1128/AAC.00517-08. 18694951 PMC2565906

[36] CLSI. 2017 Reference method for broth dilution antifungal susceptibility testing of yeast; approved standard-4th ed. Clinical and Laboratory Standards Institute, Wayne, PA.

[37] Liu Z, Myers LC 24 10 2017, posting date Mediator tail module is required for Tac1-activated CDR1 expression and azole resistance in Candida albicans. Antimicrob Agents Chemother doi:10.1128/AAC.01342-17. PMC5655045 28807920

[38] Dunkel N, Liu TT, Barker KS, Homayouni R, Morschhauser J, Rogers PD 2008 A gain-of-function mutation in the transcription factor Upc2p causes upregulation of ergosterol biosynthesis genes and increased fluconazole resistance in a clinical Candida albicans isolate. Eukaryot Cell 7:1180–1190. doi:10.1128/EC.00103-08. 18487346 PMC2446669

[39] Schmieder R, Edwards R 2011 Quality control and preprocessing of metagenomic datasets. Bioinformatics 27:863–864. doi:10.1093/bioinformatics/btr026. 21278185 PMC3051327

[40] Li H, Durbin R 2009 Fast and accurate short read alignment with Burrows-Wheeler transform. Bioinformatics 25:1754–1760. doi:10.1093/bioinformatics/btp324. 19451168 PMC2705234

[41] McKenna A, Hanna M, Banks E, Sivachenko A, Cibulskis K, Kernytsky A, Garimella K, Altshuler D, Gabriel S, Daly M, DePristo MA 2010 The Genome Analysis Toolkit: a MapReduce framework for analyzing next-generation DNA sequencing data. Genome Res 20:1297–1303. doi:10.1101/gr.107524.110. 20644199 PMC2928508

[42] Cingolani P, Platts A, Wang le L, Coon M, Nguyen T, Wang L, Land SJ, Lu X, Ruden DM 2012 A program for annotating and predicting the effects of single nucleotide polymorphisms, SnpEff: SNPs in the genome of Drosophila melanogaster strain w1118; iso-2; iso-3. Fly (Austin) 6:80–92. doi:10.4161/fly.19695. 22728672 PMC3679285

