# Supplementary figures and images for: Mutations in TAC1B: a Novel Genetic Determinant of Clinical Fluconazole Resistance in Candida auris
Source: mBio. 2020 May 12;11(3):e00365-20. doi: 10.1128/mBio.00365-20 (PMC7218281; doi:10.1128/mBio.00365-20)

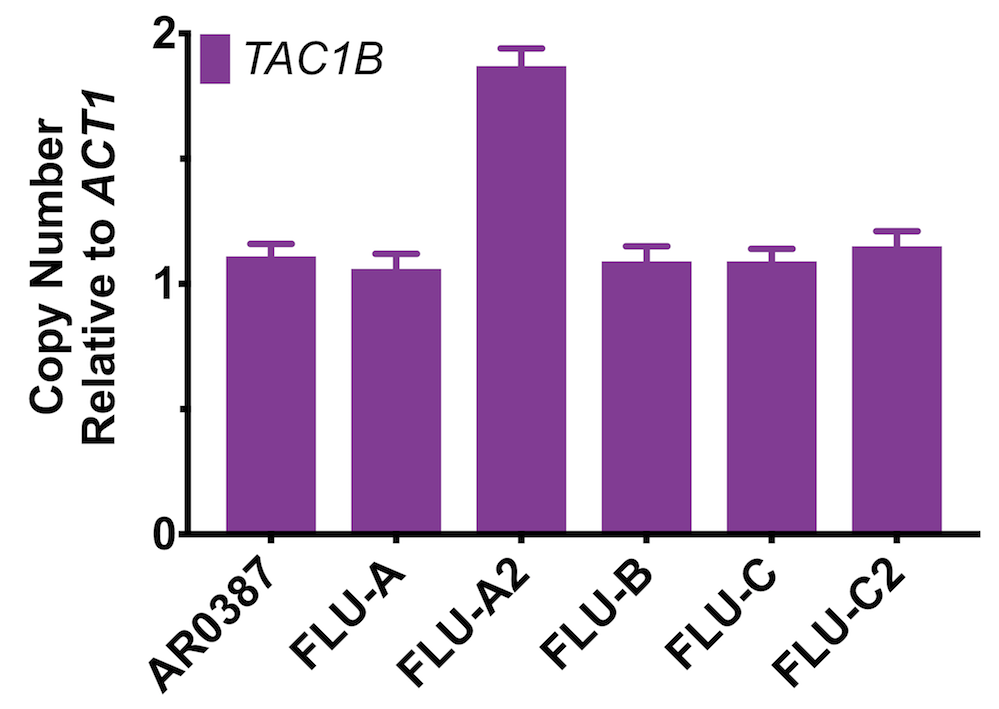

Supplement: FIG S1 [file mBio.00365-20-sf001.tif]
