# Supplementary material for: Mutations in TAC1B: a Novel Genetic Determinant of Clinical Fluconazole Resistance in Candida auris
Source: mBio. 2020 May 12;11(3):e00365-20. doi: 10.1128/mBio.00365-20 (PMC7218281; doi:10.1128/mBio.00365-20)
Supplement: TABLE S1 [file mBio.00365-20-st001.docx]

| **GENE** | **VARIANT** | **Clade I** | **Clade II** | **Clade III** | **Clade IV** | **Total** |
| --- | --- | --- | --- | --- | --- | --- |
|  |  | **(n = 126)** | **(n = 7)** | **(n = 51)** | **(n = 120)** |  |
|  |  | **%(n)** | **%(n)** | **%(n)** | **%(n)** | **n** |
| *TAC1B* (B9J08_004820) | ***A15T*** | 100(6) | 0 | 0 | 0 | 6 |
|  | S36L | 0 | 0 | 51 | 120 | 171 |
|  | S89Y | 0 | 0 | 51 | 0 | 51 |
|  | S192N | 100(1) | 0 | 0 | 0 | 1 |
|  | ***S195C*** | 100(6) | 0 | 0 | 0 | 6 |
|  | E200K | 0 | 0 | 51 | 0 | 51 |
|  | **F214S** | 0 | 50(2) | 0 | 0(1) | 3 |
|  | K215R | 0 | 7 | 51 | 120 | 178 |
|  | K225N | 0 | 0 | 51 | 0 | 51 |
|  | Q226R | 0 | 0 | 51 | 120 | 171 |
|  | **K247E** | 0 | 0 | 0 | 80(5) | 5 |
|  | I268V | 0 | 0 | 51 | 0 | 51 |
|  | D278V | 0 | 0 | 51 | 120 | 171 |
|  | Q298K | 0 | 0 | 51 | 0 | 51 |
|  | L328Q | 0 | 0 | 0 | 120 | 120 |
|  | C331S | 0 | 0 | 51 | 120 | 171 |
|  | C334F | 0 | 0 | 51 | 120 | 171 |
|  | L335S | 0 | 0 | 51 | 120 | 171 |
|  | S339A | 0 | 0 | 51 | 120 | 171 |
|  | T346I | 0 | 0 | 51 | 0 | 51 |
|  | **R495G** | 100(1) | 0 | 0 | 0 | 1 |
|  | Q503R | 0 | 0 | 51 | 0 | 51 |
|  | F580L | 0 | 0 | 51 | 0 | 51 |
|  | **A583S** | 100(5) | 0 | 0 | 0 | 5 |
|  | **P595H** | 0 | 0 | 0 | 100(1) | 1 |
|  | **P595L** | 100(1) | 0 | 0 | 0 | 1 |
|  | P607S | 0 | 0 | 0 | 0(1) | 1 |
|  | Y608H | 0 | 0 | 51 | 120 | 171 |
|  | **A640V** | 98.2(57) | 0 | 0 | 0 | 57 |
|  | Y647C | 0 | 0 | 51 | 0 | 51 |
|  | A651T | 0 | 0 | 0 | 37.5(16) | 16 |
|  | M653V | 0 | 0 | 0 | 86(7) | 7 |
|  | **A657V** | 100(15) | 0 | 0 | 0 | 15 |
|  | S754N | 0 | 0 | 51 | 120 | 171 |
|  | M809I | 0 | 0 | 51 | 120 | 171 |
|  | N773_L774del | 0 | 0 | 51 | 0 | 51 |
|  | **F862_N866del** | 0 | 0 | 0 | **97.8(46)** | 46 |
| *TAC1A* (B9J08_004819) | V13I | 0 | 0 | 51 | 120 | 171 |
|  | S116A | 0 | 0 | 51 | 120 | 171 |
|  | V145E | 0 | 7 | 51 | 120 | 178 |
|  | G149D | 0 | 0 | 51 | 0 | 51 |
|  | A288S | 0 | 0 | 0 | 120 | 120 |
|  | E313G | 0 | 3 | 0 | 0 | 3 |
|  | P371L | 1 | 0 | 0 | 0 | 1 |
|  | D500E | 0 | 0 | 51 | 120 | 171 |
|  | E560D | 0 | 0 | 51 | 120 | 171 |
|  | E565D | 0 | 0 | 51 | 0 | 51 |
|  | S627G | 0 | 0 | 51 | 0 | 51 |
|  | K713N | 0 | 7 | 0 | 0 | 7 |
|  | E758G | 0 | 0 | 51 | 0 | 51 |
|  | S762P | 0 | 0 | 51 | 120 | 171 |
|  | A766T | 0 | 0 | 51 | 0 | 51 |
