# Supplementary material for: Mutations in TAC1B: a Novel Genetic Determinant of Clinical Fluconazole Resistance in Candida auris
Source: mBio. 2020 May 12;11(3):e00365-20. doi: 10.1128/mBio.00365-20 (PMC7218281; doi:10.1128/mBio.00365-20)
Supplement: TABLE S2 [file mBio.00365-20-st002.docx]

| **Primer** | | **Sequence (5’-3’)** |
| --- | --- | --- |
| **Sequencing** | | |
|  | ERG11-SeqAmpF | GCCCTGAAAGAAACCCGTACAC |
|  | ERG11-SeqAmpR | GGCACTGGACGCAGGAAC |
|  | ERG11-SeqF1 | CCTCATCCATCGACTTGAGTTC |
|  | ERG11-SeqR1 | GGCTGGAGCTGGTTTGG |
|  | ERG11-SeqF2 | GACTGCCTTGACGAAAGAAGC |
|  | ERG11-SeqR2 | GTGTGGGTCGAACTCGTTG |
|  | ERG11-SeqF3 | GGTCAGCACACTTCAGCTTC |
|  | ERG11-SeqR3 | CTGAGCAGCGTCTCTCTTC |
|  | ERG11-SeqF4 | CGTGCAATTGGGCACC |
|  | ERG11-SeqR4 | GCCGTATTTCTCTCTGCACAG |
|  | ERG3-SeqAmpF | GTACCTGCGTCCATGTACCATG |
|  | ERG3-SeqAmpR | GGTGGTGGATCACTACGATAGCG |
|  | ERG3-SeqF1 | CACTCGGAGATCTCTGCCC |
|  | ERG3-SeqR1 | GGCTGCGAAATCGCACTG |
|  | ERG3-SeqF2 | CGTTTACACGGCCTGCAG |
|  | ERG3-SeqR2 | CAATCCACTTGTGGTGGGGC |
|  | ERG3-SeqF3 | GCCAAAGCGTTGCCCAAG |
|  | ERG3-SeqR3 | CGGTAGCGTTTTTGCTGCC |
|  | ERG3-SeqF4 | GGCTACACTGGCCTTCCG |
|  | ERG3-SeqR4 | CCAAGTGGAACCACCATGC |
|  | MRR1-SeqAmpF | GAGGCAGACGAGCGTTTTG |
|  | MRR1-SeqAmpR | GTTGGCAAAGCTGATGGG |
|  | MRR1-SeqF1 | CGAGCGGCAATTAACACTTC |
|  | MRR1-SeqR1 | CAGGGGTTAGCTAACGCC |
|  | MRR1-SeqF2 | CCAACACAACAGCAGTCC |
|  | MRR1-SeqR2 | GCTACCGCCTGGTCATCC |
|  | MRR1-SeqF3 | GGCGTGCTCTTCAGACAG |
|  | MRR1-SeqR3 | GGCATAAGCTTGCAACATCG |
|  | MRR1-SeqF4 | GGGCATGAATCGAGAGCC |
|  | MRR1-SeqR4 | CATTTCATCAGAGGTTTGCGC |
|  | MRR1-SeqF5 | GGATCATGAGAGATTGTGGCG |
|  | MRR1-SeqR5 | GAGTTAGTGATGGAACCTGACGC |
|  | UPC2-SeqAmpF | GGCAGCGCACGAACACGC |
|  | UPC2-SeqAmpR | GAGAGCGCCTTTCGTGGTGG |
|  | UPC2-SeqF1 | CTCATCCGGGAGTCTTCAG |
|  | UPC2-SeqR1 | GCGTGAGGATCGCACTAG |
|  | UPC2-SeqF2 | GTGCGGGCTTACTCAATG |
|  | UPC2-SeqR2 | CCAAACCTTGTCCTTCATCTCC |
|  | UPC2-SeqF3 | GCGTTGGTCGCCAGTGC |
|  | UPC2-SeqR3 | CTTAGATAGATGCGTTGCGCTG |
|  | UPC2-SeqF4 | CTCCATGATCGATCCGGAG |
|  | UPC2-SeqR4 | GCGGTGTCTTGTCTGGAG |
|  | TAC1A-SeqAmpF | CCCACAGGGTCAGACAGAGG |
|  | TAC1A-SeqAmpR | GGAGGCGCTTGTGTGCACG |
|  | TAC1A-SeqF1 | GGCCGTGCCTAAGCCATC |
|  | TAC1A-SeqR1 | CCAAGGGGAAGGTGGTAC |
|  | TAC1A-SeqF2 | CGCAGCTCCAGCGGTCTAC |
|  | TAC1A-SeqR2 | CCCTCCACGATCGAAATGC |
|  | TAC1A-SeqF3 | GGTCCCCGCCATCAGATAC |
|  | TAC1A-SeqR3 | GGAGGCAAAGTCCTTCACG |
|  | TAC1A-SeqF4 | CAACAGGCTCCCGCTGATG |
|  | TAC1A-SeqR4 | CTCCGAGCCGTCATTGAC |
|  | TAC1A-SeqF5 | GCCAGAGGTGGTGCCTTC |
|  | TAC1A-SeqR5 | CTCGTCCTCCTCCTCACTC |
|  | TAC1B-SeqAmpF | CGCCTCACACAAAACTTCG |
|  | TAC1B-SeqAmpR | GAAAGCCAATGCGCAGTTGG |
|  | TAC1B-SeqF1 | CAGCCATCGGGCAGTGCG |
|  | TAC1B-SeqR1 | CAGAGCAATGTCGCAGCG |
|  | TAC1B-SeqF2 | GGACCCACTCGTCGCTGC |
|  | TAC1B-SeqR2 | GCGAACCATTGGGCGTG |
|  | TAC1B-SeqF3 | GAAGGCATGGCTACTGTGC |
|  | TAC1B-SeqR3 | GCTGGAAGAAGTAGGCAAGC |
|  | TAC1B-SeqF4 | CCTTCGCAGCTCGACCCC |
|  | TAC1B-SeqR4 | GGTGGTTTCCAAATGGACCAC |
|  | TAC1B-SeqF5 | CGACGACGACCTATTGCTC |
|  | TAC1B-SeqR5 | GTCCTCTGGCTTGAGCCTG |
| **RTqPCR** | | |
|  | ACT1-F | GAAGGAGATCACTGCTTTAGCC |
|  | ACT1-R | GAGCCACCAATCCACACAG |
|  | ERG11-F | GTTTGCCTACGTGCAATTGG |
|  | ERG11-R | GTAGTCGACTGGTGGAAGCG |
|  | CDR1-F | GAAATCTTGCACTTCCAGCCC |
|  | CDR1-R | CATCAAGCAAGTAGCCACCG |
|  | MDR1-F | GAAGTATGATGGCGGGTG |
|  | MDR1-R | CCCAAGAGAGACGAGCCC |
|  |  |  |
| **qPCR** | | |
|  | ACT1-F1 | GAAGGAGATCACTGCTTTAGCC |
|  | ACT1-R1 | GAGCCACCAATCCACACAG |
|  | ERG11-F1 | GTTTGCCTACGTGCAATTGG |
|  | ERG11-R1 | GTAGTCGACTGGTGGAAGCG |
|  | ERG11-F2 | GGACTGCATCGTCGATGTTG |
|  | ERG11-R2 | GGACTGCATCGTCGATGTTG |
|  | ERG11-F3 | CACTTGCCCTTGCCTGC |
|  | ERG11-R3 | CCTGTCAGGAACGATGTCACC |
|  | CDR1-F1 | GAAATCTTGCACTTCCAGCCC |
|  | CDR1-R1 | CATCAAGCAAGTAGCCACCG |
|  | CDR1-F2 | CGAGGATGGCGTTGCTC |
|  | CDR1-R2 | CCTGATGAGCACCAAAACCAG |
|  | CDR1-F3 | CTACTGGCTCTTCTGTGGTCTC |
|  | CDR1-R3 | GTCATAGCAGCCGCGAG |
|  | MDR1-F1 | GAAGTATGATGGCGGGTG |
|  | MDR1-R1 | CCCAAGAGAGACGAGCCC |
|  | MDR1-F2 | GAGAGAGCTTCTTCGGCAG |
|  | MDR1-R2 | CCTGCTTCGTCTTTGCCC |
|  | MDR1-F3 | GTGGTGCTTCTGTGGGTG |
|  | MDR1-R3 | CACCAAACAAAGGGCCCG |
|  | TAC1B-F1 | CACGCCCAATGGTTCGC |
|  | TAC1B-R1 | GGGTGAAGGTGCCTCCATG |
|  | TAC1B-F2 | GGAGAACAACCGTGTGCTC |
|  | TAC1B-R2 | GTAATCACGTCCAGCAGCG |
|  | TAC1B-F3 | CCATGCCTATCGAGCAGC |
|  | TAC1B-R3 | GCACAGTAGCCATGCCTTC |
|  |  |  |
| **PCR** | | |
|  | TAC1B-AMP-F | TCACCGCGGCATCATCCCCACCGTTGCC |
|  | TAC1B-AMP-R | TCACGGCCGCATCATCGTTAAAATCGTGGTAAGCATAC |
|  | TAC1B-SCN-F | CATCATCCCCACCGTTGCC |
|  | TAC1B-SCN-R | GAAAGCCAATGCGCAGTTGG |
|  | TAC1B-RT-F | GACAGCGCAAGAACTATACTTCATC |
|  | TAC1B-RT-R | CAAAGAAAGTCAACATGTTGATTGCTGCTGGCGTAATTCGTACTCGTTAGGGGCGAATTGGTACCGGG |
| **crRNA target sequence** | | |
|  | TAC1B-5’-crRNA | TCTCGTTCTTCGCCATGAAC |
|  | TAC1B-3’-crRNA | TTCGTACTCGTTAGCATATG |
|  | | |
